# LET’S GET PHYSICAL: ACTIVITY, FUNCTION, AND FITNESS AS KEY COMPONENTS OF AGING AND HEALTH

**DOI:** 10.1093/geroni/igae098.1194

**Published:** 2024-12-31

**Authors:** Jennifer Schrack, Amal Wanigatunga, Adam Spira

**Affiliations:** Chair; Co-Chair; Discussant

## Abstract

Physical activity, physical function, and physical fitness (“activity, function and fitness”) are established predictors of health and longevity. Yet, many aspects of how and why measures of activity, function, and fitness are so strongly linked with physical and cognitive health in older adults remain under-addressed. This is further complicated by the multidimensional aspects of activity, function, and fitness, allowing them to function as both predictors and outcomes of health. This symposium will focus on quantifying and defining measures of activity, function, and fitness and their associations with various features of physical and cognitive health across several large U.S. cohort studies. Dr. Davoudi will present the prevalence of impairments in motor function (gait speed, chair stands, standing balance, grip strength) in the National Health and Aging Trends Study (NHATS). Dr. Wanigatunga will describe new methodology to assess the combined contribution of sensory and motor functioning to cognitive health in the Baltimore Longitudinal Study of Aging (BLSA) and the Atherosclerosis Risk in Communities (ARIC) study. Dr. Dougherty will present the association of physical activity with Alzheimer’s biomarkers (amyloid, total tau, phosphorylated tau) and cognitive functioning in the Biomarkers of Cognitive Decline Among Normal Individuals (BIOCARD) study. Dr. Twardzik will describe trends in public transit use and associations with physical activity in NHATS. Finally, Dr. Yue will present the association of sleep quality and quantity with measures of fitness and energetic efficiency in the BLSA. Collectively, these presentations will highlight novel pathways through which activity, function, and fitness contribute to healthy aging.

